# Chemical Composition and Evaluation of Insecticidal Activity of *Seseli bocconei* Essential Oils against Stored Products Pests

**DOI:** 10.3390/plants11223047

**Published:** 2022-11-10

**Authors:** Alessandro Vaglica, Ezio Peri, Natale Badalamenti, Vincenzo Ilardi, Maurizio Bruno, Salvatore Guarino

**Affiliations:** 1 Department of Biological, Chemical and Pharmaceutical Sciences and Technologies (STEBICEF), University of Palermo, Viale delle Scienze, 90128 Palermo, Italy; 2 Department of Agricultural, Food and Forest Sciences (SAAF), University of Palermo, Viale delle Scienze, Building 5, 90128 Palermo, Italy; 3 Centro Interdipartimentale di Ricerca “Riutilizzo Bio-Based Degli Scarti da Matrici Agroalimentari” (RIVIVE), Università di Palermo, Viale delle Scienze, 90128 Palermo, Italy; 4 Institute of Biosciences and Bioresources (IBBR), National Research Council of Italy (CNR), Corso Calatafimi 414, 90129 Palermo, Italy

**Keywords:** *Seseli bocconei* Guss., apiaceae, rice weevil, cowpea weevil, germacrene D, pyrethrum, LT50

## Abstract

In this study, the chemical composition of the essential oils (EOs) obtained from different aerial parts (flowers, leaves, and stems) of *Seseli bocconei* Guss., a wild species endemic of Sicily, was investigated. Furthermore, the EOs’ biocidal effects towards two pests of stored products, *Sitophilus oryzae* and *Callosobruchus maculates*, were evaluated. This activity was evaluated in Petri dish bioassays to establish the survival rate of adults treated with the EOs comparing them with solvent and a commonly used insecticide (pyrethrum). The data obtained from the toxicity bioassay evidenced that stems’ EOs and leaves’ EOs have a contact/fumigation effect towards the two insect species tested, while the EOs from the flowers did not exhibit a different mortality than the solvent. The EOs from the stem and leaves of *S. bocconei*, tested at 10 mg/petri dish, determined a LT50 of 53.38 and 42.97 h, respectively, on *S. oryzae* adults, and of 45.23 and 42.97 h, respectively, on *C. maculatus* adults. The promising bioactivity of *S. bocconei* leaves’ EOs and stems’ EOs toward *S. oryzae* and *C. maculatus* is encouraging in the perspective to test these oils and their main constituents for further experiments in the laboratory and field.

## 1. Introduction

The use of essential oils (EOs) as an alternative to synthetic insecticides has been growing in the last years, because of their wide bioactivities and high biodegradability [1,2]. EOs are mixtures of hundreds of metabolites with several properties determined by single or synergistic actions, as they exert acute toxicity, antifeedant activity, oviposition deterrence, repellence, and attraction [3,4]. Few studies indicated that, although the mechanisms of action are not perfectly known, the toxicity effect of oils, is often determined by the interaction of the EO with the insects’ nervous system and the inhibition of acetylcholinesterase (AchE), or by the antagonism of octopamine receptors [5]. However, other types of insecticidal mechanisms such as antifeedant/growth inhibition, suppression of reproductive behavior, and reduction of fecundity and fertility have been described [6,7,8,9].

In consideration of their relatively low toxicity toward humans, the use of EOs can be particularly useful as alternative control method for its application in food storages and warehouses where stored product pests can determine the spoilage and losses of the product with resulting remarkable economic impacts in terms of quality and market access [10,11]. The control of these pests in past decades mainly relied on the use of synthetic contact insecticides [12], that with time showed negative aspects as increasing resistance phenomena [13] and had strong impacts on the environment and human health [14,15]. The use of EOs as alternative control method is suggestable also in consideration of the increasingly stringent environmental regulations on the use of insecticides worldwide, aimed to reduce the use of synthetic chemicals in favor of other approaches, which are ecologically more sustainable, such as the ones centered on natural products [16]. In countries as China, Iran, Turkey, and India that have more relaxed regulatory requirements on the use of such botanicals in comparison with the EU and USA, these aspects have determined a growing interest in research concerning the possible use of plant extracts with insecticidal properties [17,18].

Despite this growing interest from research and the increasing use of EOs as alternative tools for pest management, still many valuable wild plant species and their metabolites are unexplored; therefore, it is important to conduct more studies on their possible deterrent and insecticidal properties [19]. Furthermore, many wild plants species are growing in marginal lands and their cultivation could be carried out in such contexts, where other cultivated species cannot be accomplished. Among these, species of the Apiaceae family, due to their availabilities and high EOs yields, play an important role in this sense. The Apiaceae is a family of flowering plants, which includes 3780 species divided into 434 genera and distributed from the northern temperate regions to the high altitude counties in tropical areas. Despite the different subdivisions on the territory, the plants belonging to this family have in common inflorescences consisting of simple or compound umbels, aromatic herbaceous natures, and sunken stems [20]. In addition to their anticancer, analgesic, antimicrobial, and diuretic activities, the essential oils from plants of the Apiaceae family have recently shown promising potential as insecticides, especially thanks to their natural origin, ease of production, safety, and efficacy. New strategies are being developed to improve the physicochemical properties of EOs as well as their biological activities, in such a way as to increase their use as natural insecticides. Some of the main applications which highlight the use of Apiaceae plants’ essential oils and extracts in immature mosquito populations are [21]: for example, the EO of *Carum copticum* L. was highly effective against different targets, such as the larvae of *Tuta absoluta* (Meyrick) (Lepidoptera: Gelechiidae) [22], or the anise EO that has shown high toxicity against *Leptinotarsa decemlineata* Say (Coleoptera: Chrysomelidae) (Skuhrovec et al., 2020) [23]. Other Apiaceae species whose essential oils have been shown to be particularly active as insecticides are *Crithmum maritimum* L. [24], *Sison amomum* L., *Echinophora spinosa* L., *Heracleum sphondylium* L. subsp. *sphondylium*, *Heracleum sphondylium* subsp. *ternatum* (Velen.) Brummit, *Trachyspemum ammi* (L.) Sprague [25], and *Ridolfia segetum* (L.) Moris, an ancient food plant, utilized both in Sicily and Morocco [26].

*Seseli* L., belonging to the Apiaceae family, is a genus distributed in Europe, Africa, Asia, North America, and Australia [27] that counts thirty-four species for Europe in the Flora Europea [28]. This genus consists of aromatic herbs and economically important species used as food, spices, condiments, and ornamentals [29]. Moreover, thanks to its abundance in linear and angular pyranocoumarins, many species of this genus showed various health effects such as antifungal, antibacterial, anti-inflammatory, antinociceptive, antitumor, anti-rheumatic activities, and protective effects on human lymphocytes’ DNA [30,31,32,33,34,35]. In Sicily, only three *Seseli* taxa are reported and studied mostly for their EOs: *S. bocconei* Guss. [36], *S. tortuosum* subsp. *Tortuosum*, and *S. tortuosum* subsp. *Maritimum* (Guss.) [37,38]. *Seseli bocconei* is an endemic species of Sicily, growing only in the north-west part of the island and in the Aegadian and Aeolian Islands on limestone cliffs near the sea (0–150 m a.s.l.). This plant is 30–60 cm high, glabrous, and glaucous. Its stems are erect, but woody at the base. The lower leaves are 1–3 times ternate, with lanceolate segments of (3–5) × (10–30) mm, at the base widened up to 10 mm, generally trifid at the apex. Umbels have a diameter of 4.5–8 mm, with 8–15 rays, glabrescent or slightly pubescent only on the internal side. The petals are white and hairless. The fruit is hairless, 2–2.5 × 4–6 mm in size, with enlarged ribs. Generally, it flourishes from September to October.

In order to explore new possible sources of active metabolites toward stored product pest species, here we evaluated the composition of secondary metabolites and the insecticidal activity of EOs obtained separately from flowers, leaves, and stems of *S. bocconei* collected in Sicily against two coleopteran species attacking stored commodities: *Sitophilus oryzae* L. (Coleoptera: Curculionidae) and *Callosobruchus maculatus* (F.) (Coleoptera: Bruchinae). The different parts from the same plant species have, in fact, different chemical compositions and consequently, different biological activities as observed in other studies [39].

*Sitophilus oryzae*, also known as the rice weevil, is a primary insect pest of cereal grains in storage including wheat, maize, and rice [40,41]. *Callosobruchus maculatus*, also known as cowpea weevil, causes severe damage to most legumes (particularly on cowpea) that appear perforated and with a lower weight [42]. Nowadays, *S. oryzae* and *C. maculatus* are pests mainly treated with synthetic insecticides worldwide, where such cereals and legumes are of primary importance. However, in several contexts, chemicals application is often not carried out correctly due to a lack of knowledge of farmers with consequent negative impacts on the health of consumers, seed germination, and beneficial insects [43]. Consequently, alternative methods of control such as plant-based extracts that are sustainable, cheap, and with low environmental impact, are strongly desired for the management of these pests.

## 2. Results and Discussion

### 2.1. Chemical Analysis of Essential Oils

Each hydrodistillation of the three different parts (flowers, leaves, and stems) of *S. bocconei* yielded pale yellow EOs. Overall, twenty different compounds were identified, eleven for flowers’ EOs (representing 95.03% of total components), fourteen for leaves’ EOs (91.07%), and twelve for stems’ EOs (91.22%). All identified compounds are listed in Table 1.

The three EOs were found to be very dissimilar with regards to their composition. In fact, flowers’ EOs were found to be much more abundant in monoterpene hydrocarbons (91.63%), with sabinene (19.22%), limonene (25.16%), and sylvestrene (33.62%) as the main constituents, while the EOs from leaves showed a greater abundance of sesquiterpene hydrocarbons (85.10%), with germacrene D (36.49%) and *δ*-cadinene (16.78%) as compounds present in greater quantities. Additionally, stems’ EOs differed from the two previously EOs mentioned, showing different quantities of monoterpene hydrocarbons (37.95%) and sesquiterpene hydrocarbons (53.27%), containing mainly sabinene (15.26%), limonene (16.80%), and germacrene D (24.53%). The data obtained were in perfect agreement with the previous article on the EOs obtained from the aerial parts of *S. bocconei*, in which the main constituents were sabinene (17.46%), sylvestrene (11.69%), and germacrene D (18.48%) [36]. The results are quite different from those obtained from the hydrodistillation of the aerial parts of plants belonging to a different sub-species, such as *Seseli bocconi* Guss. subsp. *praecox* Gamisans, harvested in Buggerru (Sardinia), in which the only component in common was sabinene (20.10%). In fact, the composition of *S*. *bocconi* subsp. *praecox* showed the occurrence of several oxygenated terpenes such as *trans-β*-terpineol (6.30%), himachalol (4.40%), and terpin-4-ol-acetate (4.10%) that are absent in EOs from *S. bocconei* [44]. The data obtained additionally showed the difference between *S. bocconei* and the other two Sicilian endemic *Seseli* species: *S. tortuosum* subsp. *tortuosum* and *S. tortuosum* subsp. *maritimum* rich of monoterpene hydrocarbons such as *α*-pinene, *β*-pinene, and *δ*3-carene. When comparing to the EOs obtained from the different vegetative parts of other plants belonging to the same genus, a substantial difference was noted. For instance, only the occurrence of germacrene D in the leaves’ and in the stems’ EOs, was in common with the EO composition of *Seseli annum* L., collected in Serbia (19.10% in the leaves and 6.90% in the stems). Instead, *Seseli rigidum* Waldst. shared only the presence of the monoterpene hydrocarbon sabinene in the flowers’ (19.80%) and in the stems’ (6.50%) EOs [45,46]. On the other hand, a certain similarity was shown with the composition of *Seseli libanotis* (L.) W.D.J. Koch (Austria): EOs from the flowers showed as main constituents sabinene (10.70%) and germacrene D (4.60%), EOs from the leaves exhibited the presence of germacrene D (11.00%), while EOs from the stems showed the occurrence of germacrene D (7.80%), sabinene (5.80%), and limonene (5.70%) as more abundant compounds [47]. *δ*-Cadinene, as a main constituent, was reported only for the EOs of the stems of *Seseli peucedanoides* (M.Bieb.) Koso-Pol (collected in Serbia) [48], while the presence of the monoterpene hydrocarbon sylvestrene was not detected in any essential oil of the genus *Seseli.* Limonene, the second most abundant component of the oil from the flowers of *S. bocconi*, has been detected in good quantity in the flowers’ oils of *S. montanum* ssp. *peixotoanum* (Samp.) M. Laínz from Portugal (7.7–8.8%) [49], *S. rigidum* from Serbia [50], and *S. buchtormense* (Fisch. ex Sprengel) W. Koch from Russia [51].

### 2.2. Toxicity Bioassay

The LT50 calculated for the *S. bocconei* stems’ EOs or leaves’ EOs toward *S. oryzae* adults was 53.38 and 42.97 h, respectively, from treatment, while the flowers’ EOs did not elicit a mortality different from the solvent. Similarly, the LT50 calculated for the *S. bocconei* stem and leaves’ EOs toward *C. maculatus* adults was 45.23 and 42.97 h, respectively, from treatment, while the flowers’ EOs did not elicit a mortality different from the solvent.

**Figure 1:**
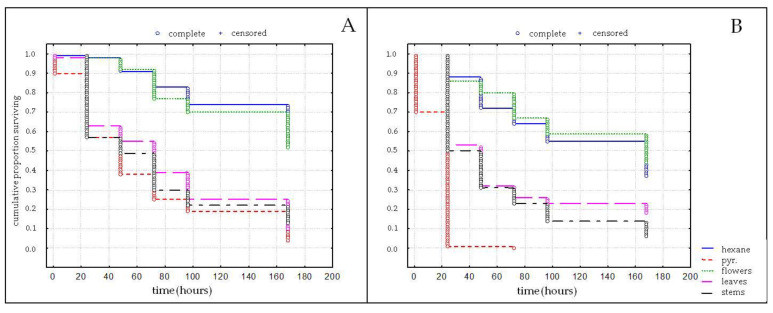
Survival rate curves of *Sitophilus oryzae* (**A**) and *Callosobruchus maculatus* (**B**) adults treated with 10% hexane solutions of EOs of *Seseli bocconei* from different parts: flowers (green line), stems (purple line), and leaves (black line). Hexane (blue line) and pyrethrum (red line) were used as a negative and positive control, respectively.

The effects of the use of *S. bocconei* aerial part EOs on the survival time of *S. oryzae* and *C. maculatus* are reported in Figure 1. Overall, in both experiments, a significant effect of the EOs tested on the mortality of both pest species was observed. In detail, *S. oryzae* evidenced highly significant differences in mortality of the adults (χ^2^ = 165.81; df = 4; *p* < 0.001), with survival rates of the individuals treated with *S. bocconei* EOs from stems or leaves similar to the ones treated with pyrethrum (Figure 1A). *Callosobruchus maculatus* adults also exhibited a different survival rate among the treatments (χ^2^ = 226.15; df = 4; *p* < 0.001), with lowest survival rates determined by pyrethrum but markedly lower survival rates of individuals treated with *S. bocconei* EOs from stems or leaves in comparison to solvent and flowers’ EOs (Figure 1).

The data obtained from toxicity bioassay evidenced that the stems’ and leaves’ EOs of *S. bocconei* have a toxicity effect towards the two insect species tested, while the EOs from the flowers did not exhibit a different mortality than the solvent. Considering the type of bioassay conducted here, it is not possible to determine whether the toxicity exhibited by the EOs is determined by contact, fumigation activity, or both. Specifically, against *S. oryzae*, the EOs from the stems and leaves exhibited a toxicity similar to the pyrethrum, and higher than the one observed in insects treated with solvent. Differently, the toxicity evidenced on *C. maculatus* adults was lower in comparison with pyrethrum, but still higher than the one determined by the solvent. The EOs from the stems and leaves of *S. bocconei* exhibited corrected mortality values after 96 h of 66.22% and 70.27%, respectively, toward *S. oryzae*, and 58.18% and 74.55%, respectively, toward *C. maculatus*. Such a kind of laboratory research, carried out bioassaying EOs as candidate tools for pest management, is an essential step for the future authorization of their use, particularly when strict regulatory regimes such as those in the EU are in force [52].

In our study, we assayed the entire EOs and not their components individually, so it is not possible to establish if the bioactivity that the EOs exhibited was exerted by some specific chemical or by the synergistic effect of the mixture. Nonetheless, we can suggest that the results obtained could be determined by the relatively high content of sesquiterpenes present in the EOs from the stems and leaves of *S. bocconei,* and the reduced abundance in flowers’ EOs. In particular, the main common sesquiterpene hydrocarbon present in both the leaves and stems was germacrene D, with percentages above 35% in the leaves’ EOs and around 25% in the stems’ EOs. In fact, several studies evidenced that EOs rich in this compound had insecticidal activity. For example, EOs from *Artemisia capillaris* (Yin-Chen) and *A. mongolica* (Fisch. ex Besser), containing 10% and 8% of germacrene D, respectively, exerted toxicity (contact and fumigant) against the maize weevil, *Sitophilus zeamais* Motchulsky (Coleoptera: Curculionidae) [53], while the EOs from *Artemisia campestris* L., containing 9% of germacrene D, showed remarkable toxicity against *Culex quinquefasciatus* Say (Diptera: Culicidae) and moderate toxicity towards *Musca domestica* L. (Diptera: Muscidae) [54]. Moreover, EOs from *Lantana camara* L. reported to have a high percentage of germacrene D [55] are toxic to *Callosobruchus chinensis* (L.) (Coleoptera: Bruchinae) [56].

In a recent study from Pereira et al. [57], nanospheres, derived from *Zanthoxylum rhoifolium* fruits’ EOs, containing 8% of germacrene D, showed promising photostability and biocidal activity versus *Bemisia tabaci* (Gennadius) (Sternorrhyncha: Aleyrodidae). Another recent study from de Oliveira et al. [58] evidenced that germacrene D deterred egg hatching and caused larval death of *Aedes aegypti* (L.) and *Ae. albopictus* (Skuse) (Diptera: Culicidae), by inhibiting the action of acetylcholinesterase, whilst also having less toxicity to non-target aquatic fauna than the synthetic insecticide temephos. In accordance with the literature, we can advise that further studies could establish the role of germacrene D in terms of its insecticidal properties toward these species.

Among the other main sesquiterpenes present in the stems and leaves of *S. bocconei* that might have elicited toxicity towards the tested insects, *γ*-muurolene and *δ*-cadinene have been found in the EOs from *Hypericum scabrum* L. (Guttiferae), exerting toxic activity to the broad bean weevil, *Bruchus dentipes* (Baudi) (Coleoptera: Bruchinae) [59]. In addition, *δ*-cadinene was observed as the main component in *Kadsura heteroclita* (Roxb.) Craib leaves’ EOs that exhibited larvicidal activity towards the mosquitoes *Anopheles stephensi* Liston, *Aeaegypti*, and *C. quinquefasciatus* [60]. In conclusion of this study, the experimental data obtained showed that the tested EOs from the leaves or stems of *S. bocconei* exhibited encouraging bioactivity towards *S. oryzae* and *C. maculatus*. The EOs tested and the main components therein, in particular germacrene D and/or the other sesquiterpenes detected in stems and leaves, could be considered as promising candidates for more trials in the laboratory and semi-field conditions, to define alternative tools that can be useful to give the opportunity to farmers and retailers to replace chemically synthetic pesticides. In this context, the production and distribution problems of plant-based pesticides are the main challenges together with a need to start an awareness campaign that discusses the use of plant-based insecticides with local people and farmers [61].

## 3. Materials and Methods

### 3.1. Plant Materials

Flowers, leaves, and stems from several individuals of *S. bocconei*, at the full flowering stage, were collected at Capo Zafferano, Palermo, Sicily, Italy, at about 14 m a.s.l., 38°06′36.27″ longitude N and 13°31′56.26″ latitude E, in October 2021. One of the samples, identified by Prof. Vincenzo Ilardi, has been stored in the University of Palermo Herbarium (No. PAL 2865/2021).

### 3.2. Essential Oils Extraction

Extraction of EOs was carried out according to Basile et al. [62]. Air-dried flowers (161.00 g), leaves (348.00 g), and stems (419.00 g) were separately hand-cut into small fragments, and then subjected to hydrodistillation for 3 h, according to the standard procedure described in European Pharmacopoeia (2020) [63]. Samples yielded 0.73%, 0.20%, and 0.13%, for flowers’, leaves’, and stems’ EOs, respectively.

### 3.3. Chemical Analysis of Essential Oils

Analysis of EOs was performed according to the procedure reported by Badalamenti et al. [64].

### 3.4. Insects

*S. oryzae* and *C. maculatus* cultures were reared at the University of Palermo (Italy), Department of Agriculture Food and Forest Science, in a climatic chamber at 25 ± 2 °C, 50–60% r.h., and with a 16:8 light:dark photoperiod. Species were reared separately in plastic cages (25 cm × 25 cm × 40 cm) with two mesh-covered holes (5 cm diameter) for ventilation and were fed with a mixture of seeds of legumes (*C. maculatus*) or with a mixture of wheat flour and rice 1:1 *w/w* (*S. oryzae*).

### 3.5. Toxicity Bioassays

Bioassays to evaluate the toxicity of *S. bocconei* flowers’ EOs, leaves’ EOs, and stems’ EOs against *S. oryzae* and *C. maculatus* were performed by using glass Petri dishes (9 cm diameter). *Seseli bocconei* leaves’, stems’, and flowers’ EOs were separately dissolved in *n*-hexane (>99%, Sigma-Aldrich, Milan, Italy) to achieve the concentration of 10%. An aliquot of 100 µL of solution was gently pipetted in the lower face of the Petri dishes to cover all the surface. After solvent evaporation (2 min), adults were placed inside the Petri dishes with 2 g of food (same used for rearing), that was immediately closed. For each replication, twenty 2–8 days old adults of *S. oryzae* or *C. maculatus* were used. Each bioassay was replicated ten times for each species and EO. As a negative control, an identical number of replications was carried out by pipetting 100 µL of *n*-hexane, while as a positive control, a 10% pyrethrum hexane solution was used. After the start of the bioassays, Petri dishes containing adults were moved into a climatic cell at 25 ± 2 °C and with a 16:8 light:dark photoperiod. Toxicity of each EO was assessed in terms of survival time by counting the dead individuals after 1, 24, 48, 72, 96, and 168 h from the start of the experiment to evaluate lethal time fifty (LT50) using probit analysis [65]. Furthermore, data obtained were analyzed using a Kaplan–Meier survival time analysis by Statistica 10.0 for Windows (Statsoft 2001, Vigonza, PD, Italy).

## Figures and Tables

**Table 1:** Chemical composition (%) of EOs of three different parts of *Seseli bocconei*: flowers, leaves, and stems.

No.	Compounds ^a^	LRI ^b^	LRI ^c^	Flowers ^d^	Leaves ^d^	Stems ^d^
**1**	*α*-Pinene ^e^	1002	1017	0.89	0.88	4.41
**2**	3-Thujene	1014	1030	5.13	-	-
**3**	*β*-Pinene ^e^	1079	1099	1.71	0.72	1.48
**4**	Sabinene ^e^	1095	1115	19.22	1.34	15.26
**5**	*α*-Phellandrene	1156	1174	0.76	-	-
**6**	*β*-Myrcene ^e^	1159	1176	1.45	-	-
**7**	*α*-Terpinene	1163	1179	3.69	-	-
**8**	Limonene ^e^	1177	1193	25.16	3.03	16.80
**9**	Sylvestrene	1194	1205	33.62	-	-
**10**	*α*-Cubebene	1442	1461	-	0.84	0.23
**11**	*δ*-Elemene	1469	1479	0.13	-	1.76
**12**	*α*-Copaene	1495	1500	-	2.07	1.78
**13**	Elixene	1497	1514	-	1.56	-
**14**	Isoledene	1499	-	-	1.50	3.09
**15**	*epi*-Bicyclosesquiphellandrene	1617	1633	-	3.64	-
**16**	*α*-Amorphene	1659	1679	-	7.30	3.21
**17**	*γ*-Muurolene	1666	1684	-	7.83	9.08
**18**	Germacrane D ^e^	1698	1706	3.27	36.49	24.53
**19**	*α*-Muurolene	1731	1740	-	7.09	-
**20**	*δ*-Cadinene	1738	1744	-	16.78	9.59
	**Monoterpene Hydrocarbons**			**91.63**	**5.97**	**37.95**
	**Sesquiterpene Hydrocarbons**			**3.40**	**85.10**	**53.27**
	**Total**			**95.03**	**91.07**	**91.22**

^a^ Metabolites listed in order of elution on an DB-Wax column; ^b^ Linear retention indices on a DB-Wax polar column; ^c^ Literature linear retention indices (https://webbook.nist.gov/; accessed on 10 October 2022); ^d^ Amounts (%) of the separated compounds calculated from integration of the peaks; ^e^ Co-injection with authentic standards.

## Data Availability

Not applicable.
